# Macromolecular crystallography beamlines at the Canadian Light Source: building on success

**DOI:** 10.1107/S2059798320007603

**Published:** 2020-06-19

**Authors:** Michel Fodje, Kiran Mundboth, Shaunivan Labiuk, Kathryn Janzen, James Gorin, Denis Spasyuk, Scott Colville, Pawel Grochulski

**Affiliations:** a Canadian Macromolecular Crystallography Facility, Canadian Light Source, 44 Innovation Boulevard, Saskatoon, SK S7N 2V3, Canada

**Keywords:** macromolecular crystallography, beamline, double crystal/multi-layer monochromator, Canadian Light Source

## Abstract

The Canadian Macromolecular Crystallography Facility (CMCF) consists of two beamlines dedicated to macromolecular crystallography: CMCF-ID and CMCF-BM. After the first experiments were conducted in 2006, the facility has seen a sharp increase in usage and has produced a significant amount of data for the Canadian crystallographic community. Upgrades aimed at increasing throughput and flux to support the next generation of more demanding experiments are currently under way or have recently been completed. At CMCF-BM, this includes an enhanced monochromator, automounter software upgrades and a much faster detector. CMCF-ID will receive a major upgrade including a new undulator, a new monochromator and new optics to stably focus the beam onto a smaller sample size, as well as a brand-new detector.

## Abstract 2

The current capabilities of and future upgrade plans for the beamlines supporting structural biology at the Canadian Light Source are described.

## 1. Introduction   

**Figure 1:**
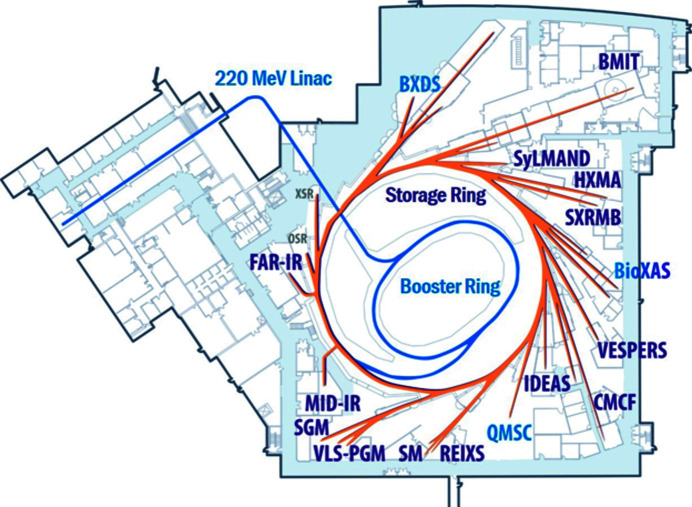
Schematic representation of the major components of the Canadian Light Source. The 2.9 GeV storage ring has a circumference of 170.88 m and currently operates with a maximum current of 220 mA. The horizontal emittance is approximately 22.7 nm rad, with a vertical emittance of 0.1017 nm rad. Operational beamlines which can support structural biology and biophysics projects include the Canadian Macromolecular Crystallography Facility (CMCF) and the BioMedical Imaging and Therapy (BMIT), Mid Infrared Spectromicroscopy (MID-IR) and Biological X-ray Absorption Spectroscopy (BioXAS) facilities.

The Canadian Macromolecular Crystallography Facility (CMCF) operates two beamlines, CMCF-ID (08ID-1) and CMCF-BM (08B1-1), at the Canadian Light Source (Grochulski *et al.*, 2011 ▸; Fodje *et al.*, 2014 ▸). Located at the southeast corner of the facility (Fig. 1 ▸), both beamlines are moving towards full automation and now operate almost exclusively through remote access. About 70 crystallography groups are supported, including Canadian, American and international groups. The facility is dedicated to providing approximately 25% of the available beamtime to commercial projects and, at least on CMCF-ID, this is fully utilized primarily through remote control of the beamline or the mail-in crystallography service, which is managed by a dedicated industrial team.

In 2016 the facility marked its tenth year of crystallo­graphy experiments, and 2017 saw the publication of the 1000th structure in the Protein Data Bank (https://www.rcsb.org/) as well as the 500th peer-reviewed publication using data collected at the facility. Now, after 13 years of successful operation, the facility is undergoing a series of upgrades aimed at increasing throughput and flux, while decreasing sample spot size, in order to support more challenging experiments that will include smaller sample sizes and serial crystallo­graphy using, for example, crystal-on-crystal chips or injectors.

## 2. CMCF-ID upgrade   

The CMCF-ID upgrade secured Canada Foundation for Innovation (CFI) funding in 2015 to increase the beam flux, reduce the beam focus size for micro-beam capabilities, improve the stability of the endstation and improve data-acquisition throughput.

**Figure 2:**
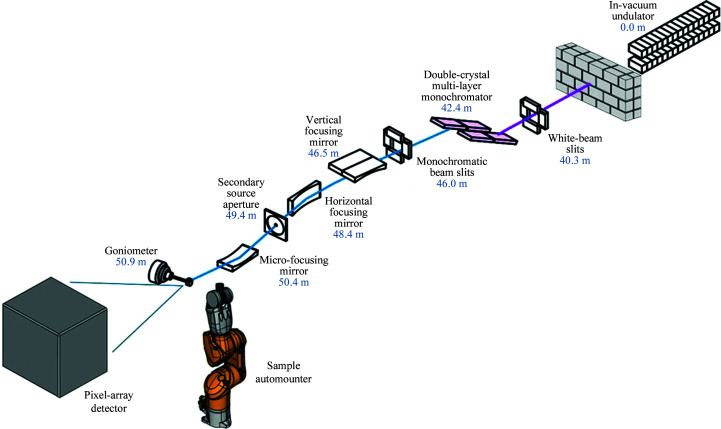
Optical layout of the planned CMCF-ID beamline. Absolute distances of the main components from the centre of the straight section of the ring are also included

**Figure 3:**
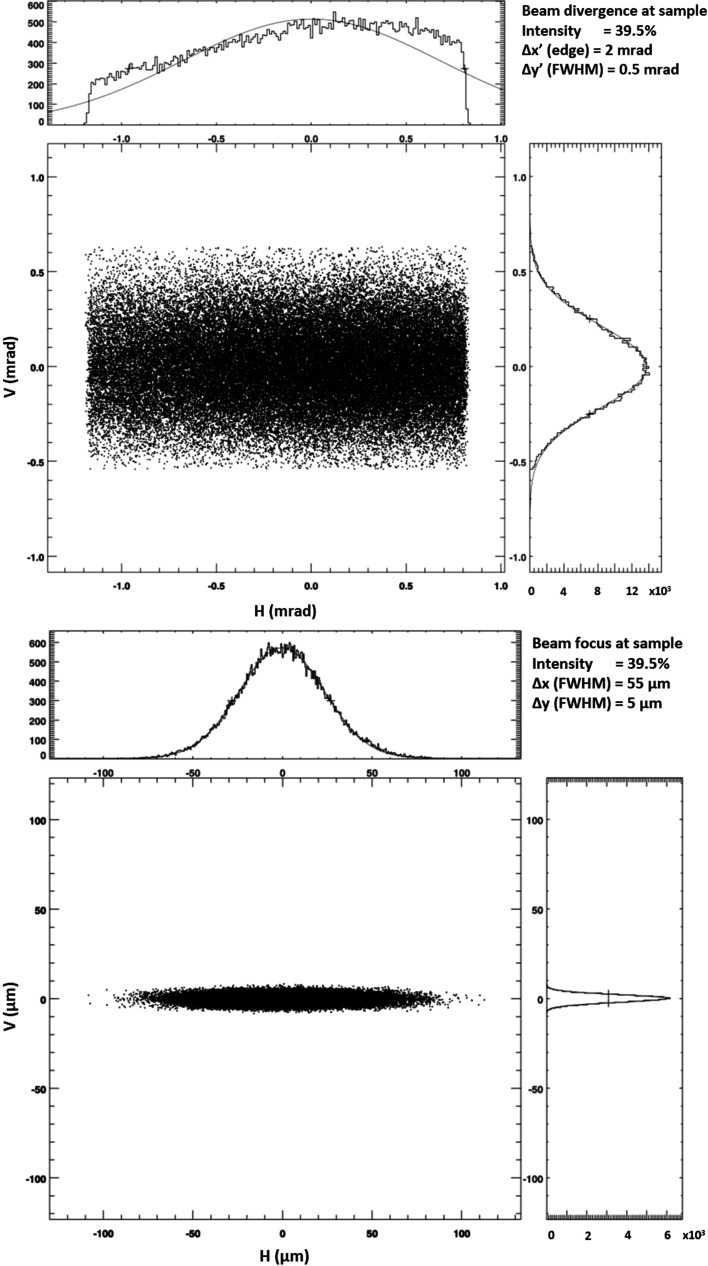
Result of ray-tracing for the CMCF-ID upgrade performed at 12 keV. Top, beam divergence; bottom, focus spot size at the sample position.

The design philosophy was dictated by the inherent limitations of the CLS storage ring, especially the very large horizontal emittance. Therefore, a horizontally focusing mirror located close to the sample position was selected in order to maximize the demagnification ratio and provide a fixed horizontal focus at the sample. In the vertical direction, a secondary source and a dynamically focusing mirror close to the sample were chosen so that a variable vertical beam size of 5–50 µm could be achieved. To significantly increase the flux density of the beamline, the 1.5 m long in-vacuum undulator (IVU) will be replaced with a longer 3.8 m IVU (Research Instruments). The width of the undulator magnets were optimized such that the effect of transverse nonlinear field roll-off seen by the electron beam is negligible. In isolation, the relatively low field and short period of the undulator, compared with the wigglers in the storage ring, will not have a detrimental focusing effect. The optical layout of the beamline will be changed and the endstation will be replaced. The new optics will feature a double-crystal/multilayer monochromator (DCMM; Axilon) and three new mirror systems (FMB Oxford). The vertically and horizontally focusing mirrors act in a Kirkpatrick–Baez (KB) configuration; however, they are located in different vacuum chambers owing to space restrictions. The third mirror can be dynamically bent to adjust the beam focus to the requested pinhole size to maximize flux. All three mirrors have rhodium/iridium coatings, except for the first mirror, which also has a silicon stripe for higher harmonic rejection. The r.m.s. slope errors are 0.37, 0.78 and 0.15 µrad, respectively. Together with the new undulator, this will enable the beamline to achieve a focus spot size of 5 × 55 µm with a photon flux of more than 10^13^ photons s^−1^ or up to 5 × 10^14^ photons s^−1^ in the double multi-layer (DMM) mode (1% Δ*E*/*E*). In the DMM mode, the tunability of the monochromator is limited to the 7.2–10.4 keV range (Table 1 ▸). The multilayer mirrors are arranged in series with the Si(111) crystals and will consist of two flat silicon substrates coated with 300 bilayers of Mo/B_4_C and bearing r.m.s. slope errors of 0.3 µrad. The new layout of the beamline is shown in Fig. 2 ▸ and the results of ray tracing are shown in Fig. 3 ▸. The configuration will allow the energy to be extended down to 5 keV, allowing the possibility of S-SAD work. A detailed technical design of the beamline and optics specifications will be published elsewhere.

The current PILATUS3 S 6M detector will be replaced with an EIGER X 9M detector, while the high-capacity ISARA automounter, which was installed in 2017, will continue to be a key component of the upgraded beamline.

In April 2019, the technical design report was completed. All major components have been procured and have either been received or are currently being received. The CMCF-ID beamline was closed for upgrades starting during the facility shutdown period in spring 2020, at which time the installation of the new insertion device and other components began. Installation and commissioning are expected to last about six months, with an anticipated return to service in January 2021. A summary of the upgrade timeline is shown in Table 2 ▸. To alleviate the loss of beam time to users during this period, most experiments will be accommodated on the CMCF-BM beamline, which has also been revamped in preparation for the increased demand.

## 3. CMCF-BM upgrade   

**Figure 4:**
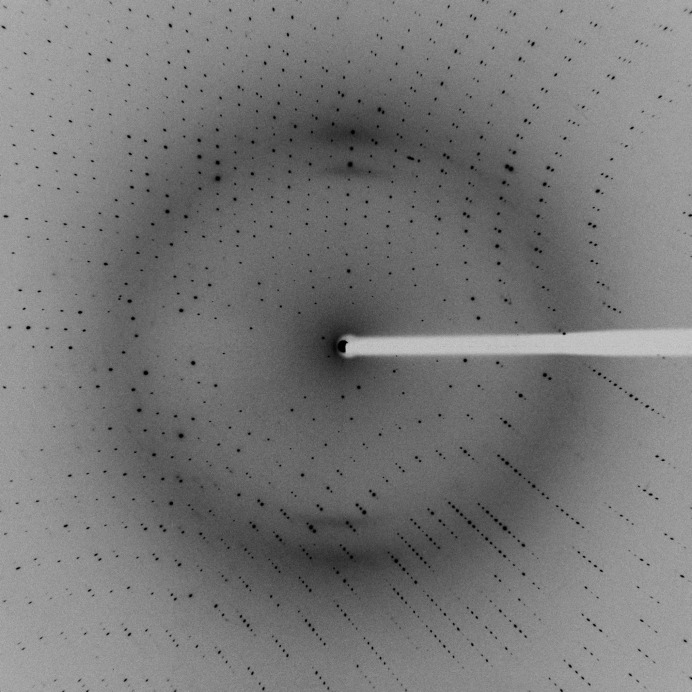
1° s^−1^ diffraction pattern from a thaumatin crystal using a 100 µm aperture in high-flux mode on CMCF-BM.

In the summer of 2019, the CMCF-BM beamline double-crystal monochromator (DCM) was upgraded to include a double multi-layer monochromator (DMM), enabling a high-flux mode. In a similar manner to the CMCF-ID beamline, the multilayers for the CMCF-BM beamline consist of two flat silicon substrates arranged in series with the Si(111) crystals and bear r.m.s. slope errors of 0.23 µrad. However, a coating of Ni_93_V_7_/B_4_C with 500 bilayers was chosen. The distances of the optical components and beamline did not change from the original design (Fodje *et al.*, 2014 ▸). The DMM operates at a fixed energy of 8.157 keV, with a 0.37% Δ*E*/*E* energy bandpass. The DMM has already been commissioned and an 18-fold increase in flux over the DCM was observed at the sample. The high-flux mode is ideal for native experiments. Experiments requiring a higher energy resolution continue to be supported with the traditional Si(111) DCM. The high-flux mode has been available for use since September 2019. An example of a 1° s^−1^ frame from a thaumatin crystal is shown in Fig. 4 ▸. The diffraction patterns are readily analysed and integrated using the standard software pipeline already available at the beamline. Initial comparisons of both modes show that similar or better quality native data can be obtained for the same crystal at the same wavelength (Table 3 ▸). A more detailed comparative analysis will be described in a future publication.

To complement the faster collection times that are now possible on the beamline, the efficiency of the Stanford Automated Mounter (SAM) has been increased to become more comparable with the ISARA automounter at CMCF-ID. The software updates on the SAM automounter now support a duty cycle of as low as 25 s (previously 2.5 min). Also, the PILATUS3 S 6M detector from CMCF-ID will be permanently moved to CMCF-BM, while CMCF-ID is equipped with the new EIGER X 9M detector.

## 4. Computing and software   

Data acquisition, automated data processing, sample information management and data access are supported through the *MxDC*, *MxLIVE* and *AutoProcess* software packages developed in-house (Fodje *et al.*, 2012 ▸). In 2019, the computing and storage infrastructure was upgraded to provide more storage capacity with faster access. Specifically, the storage server was upgraded from an 18 Tb RAID system with spinning discs connected through a 4 Gbps network to a 45 Tb SSD RAID system with 20 Gbps network access. Also, the ten-year-old 96-core compute cluster for data analysis was upgraded to more recent hardware with faster processors, providing a total of 336 cores. New versions of *MxDC* and *MxLIVE* were deployed to support the new capabilities.

Raw data collected at CMCF beamlines are stored under users’ personal accounts on a network file system and are backed up in real time on the upgraded storage server. Files are maintained at the CMCF for as long as data-storage limitations allow, which is typically up to one year. Data downloads are facilitated through users’ *MxLIVE* accounts.

Remote control of the beamlines is provided through the NoMachine client. Dedicated NoMachine servers for each beamline accept connections from a list of allowed users which is managed through *MxLIVE*. These connections provide the user with a desktop identical to that of a physical computer at the beamline, with full access to the CMCF computing infrastructure.

Demand for remote access in Canada has only grown since the first remote experiments were completed in 2011, and now accounts for about 85% of user access. Roughly 70% of remote-access experiments are completed through remote control, with the remaining 30% going through the mail-in crystallo­graphy service.

## 5. Industrial usage of the CMCF beamlines   

Over the past five years, industrial usage of the CMCF sector has seen a steady increase in the number of industrial clients from several sectors of industry. The usage of the CMCF-ID beamline has increased from 24% in 2015 to nearly 35% in 2020. A similar trend has been observed for the CMCF-BM beamline, where industrial usage grew from 5% to 11%. The majority of industrial users of the CMCF beamlines are represented by the United States (65%), followed by China (15%), Canada (10%) and the EU (10%).

Industrial access to the CMCF sector comprises two modes of access: remote control and mail-in. In 2020 nearly 55% of industry-allocated beam time was used by remote users and over 45% used mail-in access to the facility. Not surprisingly, most of the CMCF-ID allocated shifts were used by pharmaceutical industry clients, while CMCF-BM was largely used by environmental and material science clients. Of the CMCF-BM shifts used by industry, 18% were devoted to the pharmaceutical industry, 35% to environmental science and 47% to material science.

## 6. Scientific outcomes and training   

Over the years, the CMCF has seen the publication of over 700 peer-reviewed articles containing data collected at the facility, as well as over 1300 PDB depositions, 11 patents or patent applications and more than 150 Masters and PhD theses. Many high-impact scientific studies have been conducted by the CMCF user base in many areas of health research, including cancer research (Raman *et al.*, 2019 ▸; Ishizawa *et al.*, 2019 ▸), drug design (Petrilli *et al.*, 2020 ▸), antibiotic resistance (Caveney *et al.*, 2019 ▸) and antimalaria antibody research (McLeod *et al.*, 2019 ▸).

The transition from researchers having to come onsite to collect data to data collection via remote control of the beamlines has been largely facilitated by the annual CLS Mx Data Collection School. This event is hosted by CMCF staff and takes place at the Canadian Light Source, normally during a week in the spring or summer months. A series of lectures, as well as hands-on exercises, allow participants to be actively engaged in data collection while experiencing synchrotron-facility life first-hand. Crystallographers from the University of Saskatchewan support the school by mentoring participants during one-on-one data-collection sessions during their stay. Since 2010, the school has played a key role in the training of over 200 new crystallographers and has provided an opportunity for aspiring crystallographers to meet and establish new connections that are important for their careers.

## 7. Other beamlines   

Other beamlines at the Canadian Light Source that support structural biology projects include the Mid Infrared Spectromicroscopy (Mid-IR; May *et al.*, 2007 ▸), Biological X-ray Absorption Spectroscopy (BioXAS; Grochulski *et al.*, 2017 ▸) and Biomedical Imaging and Therapy (BMIT; Wysokinski *et al.*, 2013 ▸) beamlines. The Mid-IR beamline is a state-of-the-art Fourier-transform IR spectrometer and microscope which provides diffraction-limited spatial resolution to an ever-widening range of infrared spectromicroscopy experiments. The BioXAS beamline can perform extended X-ray absorption fine structure (EXAFS) experiments on 3–4 p.p.m. solid samples and millimolar solutions, and also supports multi-resolution X-ray fluorescence imaging on biological samples. Additionally, BMIT can perform X-ray computed tomography (CT) measurements at various spatial and temporal scales. Phase-enhanced imaging, multi-contrast imaging and *K*-edge subtraction and spectral imaging are also supported.

## 8. Conclusion   

We here report on recent advances at the macromolecular crystallography beamlines of the Canadian Light Source. Since 2006, the CMCF has become an integral part of the Canadian macromolecular crystallographic community, playing an important role in the development of a new generation of crystallographers through its annual CLS Mx Data Collection School, with over 200 trainees so far and over 150 graduate-level theses. The facility has also attracted the attention of industry, successfully retaining a client base that routinely makes use of up to 30% of available beamtime. The upgrade of CMCF-BM is complete and has enabled a sixfold decrease in the automounter duty-cycle time and an 18-fold increase in the photon flux at 8.15 keV using multi-layer optics. Initial data sets revealed that despite the higher energy bandwidth, the multi-layer optics produce similar or better quality data sets when compared with the Si(111) optics at the same energy. The CMCF-ID upgrade is still in progress but is expected to be completed by 2021. The updated CMCF-ID will enable high-flux micro-beam capabilities at the Canadian Light Source and these developments will allow the facility to effectively handle more challenging samples from a growing Canadian structural biology community.

## Figures and Tables

**Table 1:** Specifications of the CMCF beamlines before and after the planned upgrades Flux estimates are shown for a ring current of 220 mA.

	Current CMCF-ID	Planned CMCF-ID	Current CMCF-BM
Spectral range (keV)	6.5–18.0	5.0–20.0	5–20
Energy bandwidth (Δ*E*/*E*)			
Si(111)	∼1.5 × 10^−4^	∼1.5 × 10^−4^	∼1.5 × 10^−4^
DMM		∼1 × 10^−2^ (7.2–10.4 keV)	3.7 × 10^−3^ (8.1 keV)
Flux on the sample (photons s^−1^)			
At 12 keV	2 × 10^12^ (100 µm)	>1.5 × 10^13^ (100 µm)	>1.5 × 10^11^
	1 × 10^12^ (50 µm)	>1 × 10^13^ (50 µm)	
	5 × 10^11^ (20 µm)	>5 × 10^12^ (20 µm)	
	2 × 10^10^ (5 µm)	>1 × 10^12^ (5 µm)	
		∼5 × 10^14^ (50 µm) DMM	
At 8.1 keV			>1.5 × 10^11^ Si(111)
			>2.5 × 10^12^ DMM
Focal size at 12 keV (µm × µm)	150 (H) × 30 (V)	55 (H) × 5 (V)	210 (H) × 190 (V)
Beam crossfire at the sample at 12 keV (no pinhole) (mrad × mrad)	0.9 (H) × 0.2 (V)	1.82 (H) × 0.34 (V)	1.95 (H) × 0.30 (V)

**Table 2:** Timeline for the CMCF beamline-upgrade projects

	CMCF-BM	CMCF-ID
2017	*MxDC* and *MxLIVE* updates	*MxDC* and *MxLIVE* updates
		ISARA automouter installed
2018		Preliminary design report completed
2019	SAM performance upgrades	All major components procured
	DCMM monochromator upgrade	Delivery of new IVU, EIGER detector and MD2S microdiffractometer endstation
2020	Installation of PILATUS S 6M detector	Shutdown for installation and commissioning
2021		Full user operations

**Table 3:** Data-collection statistics for a thaumatin test crystal comparing the DCM [Si(111)] and DMM (multi-layer) modes Owing to the much higher flux for the DMM mode, the lowest available transmission of 10% was used for comparison. Statistics for the highest resolution shell are shown in parentheses. The data were processed using *XDS* (Kabsch, 2010 ▸) and data quality was assessed using *Phenix* (Liebschner *et al.*, 2019 ▸).

	Si(111) mode	Multi-layer mode
Wavelength (Å)	1.52	1.52
Data collection	720 frames at 0.5° per 0.5 s	720 frames at 0.5° per 0.5 s
Beam properties	100 µm aperture, 100% transmission	100 µm aperture, 10% transmission
Resolution range (Å)	9.948–2.100 (2.174–2.100)	9.996–2.100 (2.174–2.100)
Space group	*P*4_1_2_1_2	*P*4_1_2_1_2
Unit-cell parameters (Å, °)	*a* = *b* = 57.97, *c* = 150.48, α = β = γ = 90	*a* = *b* = 57.66, *c* = 149.69, α = β = γ = 90
Total reflections	370297 (35371)	367652 (35140)
Unique reflections	13558 (1318)	13352 (1297)
Multiplicity	27.3 (26.8)	27.5 (27.1)
Completeness (%)	98.53 (99.01)	98.62 (100.0)
Mean *I*/σ(*I*)	20.06 (3.31)	26.96 (10.18)
Wilson *B* factor (Å^2^)	30.13	23.17
*R*_merge_	0.1647 (2.326)	0.1217 (0.8305)
*R*_meas_	0.1678 (2.372)	0.124 (0.8465)
*R*_p.i.m._	0.03203 (0.4588)	0.02355 (0.1625)
CC_1/2_	0.997 (0.928)	0.998 (0.986)
